# Acute and sub-chronic toxicity study of novel polyherbal formulation in non-alcoholic fatty liver using Wistar rats

**DOI:** 10.2144/fsoa-2023-0118

**Published:** 2024-05-20

**Authors:** Anuragh Singh, K Ilango

**Affiliations:** 1 Department of Pharmacology, SRM College of Pharmacy, SRM Institute of Science & Technology, Kattankulathur – 603 203, Chengalpattu (Dt), Tamil Nadu, India; 2 Department of Pharmaceutical Quality Assurance, SRM College of Pharmacy, SRM Institute of Science and Technology, Kattankulathur – 603 203

**Keywords:** acute toxicity, NOAEL, non-alcoholic fatty liver, PHF tablet, sub-chronic toxicity

## Abstract

**Aim:** This study assessed the acute and sub-chronic toxicity of a novel polyherbal formulation tablet in Wistar rats **Materials & methods:** Acute toxicity and sub-chronic toxicity was assessed following OECD (Organisation for the Economic Co-operation and Development) guidelines based on 423 and 408. **Results & conclusion:** No mortality and toxicity showed in rats during acute toxicity. The LD_50_ of the extract was at 2000 mg/kg. In sub-chronic study, both sex rats were orally administered at 250, 500,1000 and 2000 mg/kg for 90 days and revealed no significant difference (p < 0.05) in hematological and other parameters compared with the control. Histopathology study did not reveal morphological alteration. The No observed adverse effect level of the tablet was observed until 2000 mg/kg.

## Plain language summary

Non-alcoholic fatty liver disease (NAFLD) affects around 25% of individuals globally and has become the most common long-term liver problem. The reasons why people get this disease can be different for each person. By studying natural substances, scientists have discovered that some compounds help treat the disease some of these substances can also be harmful. By studying natural substances, scientists have discovered that some compounds help treat the disease some of these substances can also be harmful. People are also trying out traditional medicines more and more, and we need to make sure they're safe. To determine whether a medication is secure, we conducted experiments in accordance with the OECD guidelines. One test examines whether a high dose of the drug is lethal. The goal is to determine the optimal dose, which is neither too low nor too excessive. Another test investigated what happens if these rats take the medicine every day for a long time. Variables such as blood tests and tissue samples are collected to make sure the medicine does not make the rats sick. In this case, we tested a medicine called a ‘PHF tablet’ for 90 days, and it didn't make the animals sick. They found that you can take a relatively high dose without any adverse effects.

## Abstract (plain language summary)

The polyherbal formulation tablet is a combination of five different herbal ingredients namely *Andrographis paniculata, Phyllanthus niruri, Terminalia arjuna, Glycyrrhiza glabra*, and *Plumbago indica.*

Acute toxicity studies demonstrate that there was no change in behavioral parameters in female wistar rat and no significant change in body weight.

NOAEL was found to be greater than 2000 mg/kg body weight.

In the sub-chronic toxicity study, PHF showed mild changes in the level of RBC and MCV compared with other parameters.

The results also showed slight changes in ALP level in liver function test.

There was non-significant changes in food and water intake.

No abnormality was observed in any of the vital organs.

Since the 1970s, non-alcoholic fatty liver disease (NAFLD), which affects around 25% of individuals globally, has become the most common chronic liver disease [1]. It is also recognized to be intimately related to components of the metabolic syndrome [2]. Despite the fact that less than 10% of people with NAFLD have liver-related complications, identifying those who are most at risk among the large population of NAFLD patients is a substantial problem [3]. Asia now has a prevalence of NAFLD of about 25%, comparable to many Western countries. Although they are becoming more common, severe liver diseases driven by NAFLD and hepatocellular cancer are still uncommon. Around 8–19% of Asians with body mass indices under 25 kg/m^2^ have this condition, which is sometimes referred to as ‘lean’ or ‘non-obese’ [4]. Environmental elements, genetic predispositions and excessive dietary intake all contribute to the emergence of metabolic liver disease. Excessive dietary fat intake, particularly triglycerides, sphingolipids and phospholipids, leads to hepatocyte steatosis. Free fatty acids (FFAs), mostly from adipose tissue lipolysis but also from dietary fat and *de novo* lipogenesis, are used to make hepatic triglycerides: The three primary sources of fatty acids (FAs) are chylomicrons (15%), lipolysis of adipose tissue (59%) and *de novo* lipogenesis (26%) [5]. Due to its high incidence, it is now the most quickly increasing cause of liver-related death worldwide. It is also a substantial contributor to end-stage liver disease, primary liver cancer and liver transplantation, placing a tremendous financial strain on the healthcare system [6,7]. Despite growing concern, few government initiatives or regulations address this chronic severe illness [8]. Several clinicopathological disorders are grouped under the ‘NAFLD’ umbrella [9]. Histologically, steatosis with or without moderate inflammation characterizes non-alcoholic fatty liver (NAFL), and a necro-inflammatory variant (NASH) is further illustrated by the presence of hepatocellular injury (hepatocyte ballooning) [10]. The primary disease-causing variables may differ significantly across NAFLD patients [11]. In addition, there are variations in how diseases progress and how well they respond to treatment. Assessing the severity of liver disease and providing prognostic information requires information on disease activity and, in particular, the extent of liver fibrosis [12]. The increasing knowledge from metabolomics, genetics, and other domains may enable illness phenotyping and future disease stratification [13]. *In silico* methods that can aid in the prediction of *in vivo* outcomes (i.e., LD_50_) are analysed, and it is concluded that predictions obtained using *in silico* approaches are now suitable for supporting assessment of LD_50_-based acute toxicity for GHS classification [14,15]. The evaluation of chemical toxicity is essential for determining the negative effects these substances may have on humans, animals, vegetation and the surrounding ecosystem. In addition, it is a fundamental component of the drug design procedure. Utilizing animal models for toxicity testing is a time-honored practise. However, *in vivo* animal experimentation is restricted due to time constraints, ethical concerns and financial constraints [16]. Hence, the use of computational methodologies in assessing the toxicity of substances is well acknowledged. In the field of toxicology, *in silico* approaches are used to evaluate the toxicity of substances via the utilisation of computer tools for analysis, simulation, visualisation, and prediction [17]. The field of *in silico* toxicology seeks to enhance the current repertoire of toxicity tests by using computational models and simulations to forecast toxicity, prioritise substances for further evaluation, provide guidance for toxicity testing, and reduce the occurrence of late-stage failures in the creation of pharmaceutical medications [18].

Each herbal component of the polyherbal formulation (PHF) tablet has been shown to have hepatoprotective properties in published research. The Indian system of medicine makes considerable use of *A. paniculata* (Acanthaceae), often known as the ‘king of bitters’, either by itself or in conjunction with other herbs in a variety of hepatoprotective formulations [19]. Andrographolide, the primary active constituent of *A. paniculata* and a diterpenoid lactone, is chiefly responsible for the antihepatotoxic activity of the herb in different animal models [20]. Other diterpenes, including andrographiside and neo-andrographolide, significantly increased cellular antioxidant components while lowering lipid peroxidation in mice when administered. The positive effects of silymarin on liver functions were shown to be equal to the hepatoprotective qualities [19]. Herbal constituents such as alkaloids, anthocyanins, flavonoids, glycosides, phenols, resins, saponins and tannins have diverse biological and pharmacological effects. It has been demonstrated that these bioactive compounds have the potential for both therapeutic benefit and toxicity. The nature, quantity, and potency of the plants' bioactive components have been linked to their toxicity [21]. The use of traditional medicine is expanding globally, and public health and safety issues are also receiving increased attention.

This study is primarily focused on to verify any toxicity being produced from the prepared polyherbal tablet and also to evaluate any abnormal changes in the behavior, and biochemical parameter in the rat consuming the drug for a period of 90 days. This formulation has not been scientifically prepared and tested yet in the formulated form for the treatment of NAFLD. There are no standard medicine that has been approved by FDA for the treatment of NAFLD. Very few papers explain the safety evaluation of specific plant constituents carried out in compliance with generally accepted testing methodologies. However, the aforementioned medicinal plants are available with considerable pharmacological information on liver function. In addition to the positive benefits shown, producing detailed toxicological data on the test material is crucial to ensure its safety when used, especially for extended periods. To define the no observable effect level (NOEL) and determine the safety of PHF tablets for use as a hepatoprotective medication, we thus conducted acute and sub-chronic oral toxicity experiments in rats in the current study [22].

## Materials & methods

### Investigational herbs

Combining herbal powders based on their therapeutic index is necessary to create traditional medicines for polyherbal formulations. Following a thorough literature review, five distinct herbal plants were chosen with the help of an Ayurvedic and Siddha specialist, and formula was created for the tablet formulation. Prof P Jayaraman (late), a phytochemist from the Plant Anatomy and Research Centre, authenticated the plants *Andrographis paniculata* (PARC/2021/4525), *Phyllanthus niruri* (PARC/2021/4526), *Terminalia arjuna* (PARC/2021/4535), *Glycyrrhiza glabra* (PARC/2021/4527) and *Plumbago indica* (PARC/2021/4536). For future use, the authenticated specimen is submitted to the Department of Pharmacognosy. Following validation, the entire plant was meticulously cleaned and allowed to air dry in the shade. Using a porcelain mortar and pestle, the dried leaves were removed from the stalks and pounded to a coarse powder. The powder was then measured at 500 g dry weight, steeped in a solution of 70% ethanol and 30% distilled water, and put through a soxhlet process for 72 h at 60–70 °C. To get the extract powder, the filtrate was concentrated and evaporated at 40 °C under reduced pressure using a rotary evaporator.

### Polyherbal formulation tablet

The plants were selected in the following range, *Andrographis panicul*ata in the range of 5–50%; *Glycyrrhiza glabra* in the range of 1–50%; *Plumbago indica* in the range of 3–50%; *Terminalia arjuna* in the range of 5–50%; *Phyllanthus niruri* in the range of 10–65%; and pharmaceutically acceptable excipients. The polyherbal formulation (PHF) was punched into tablets. The tablet was prepared in bulk and stored in an air tight container under room temperature. Accelerated stability studies was also performed for the tablet for a period of one year to check the stability. The quality control for the PHF tablet was tested, so that the tablet can be qualified for further studies. The corresponding formulation was patented and published in Indian Intellectual property under patent no. 202341030818**.** Depending on the animal's weight, tablets were ground and put into a suspension by adding distilled water. With a volume of 10 ml/kg, only fresh suspension was administered to the rat orally to preserve the stability of the preparation.

### Experimental animals

After receiving consent from the Institutional Animal Ethical Committee (IAEC) from the SRM College of Pharmacy, SRM Medical, and Health Science with approval number (IAEC/256/2021), the Wistar rats were procured from Biogen Laboratory Animal Facility, reg. no. 971/PO/RcBIBt/S/2006/CPCSEA. The animals weighed between 100 and 150 g at the time of procurement. The experiments were carried out as per the OECD recommendations and Good Laboratory Practice. They were kept in cages with wire net coverings in a room with a 12 h light/dark cycle, 70% humidity, 23–25 °C temperature, and enough ventilation. All animals had unlimited access to food and water. For the purpose of acclimation and to reduce the entry of pathogens into the colony, animals were isolated for a week. All animals were weighed when the quarantine period was through, given a special identification number for each group, and placed in cages according to their groupings.

### Acute oral toxicity

OECD guideline no. 423 was followed in the acute toxicity investigation. Three animals of the same sex are used in each phase of the acute toxic class technique (1) described in this Guideline. For a decision to be made on the acute toxicity of the test chemical, an average of 2–4 steps may be required, depending on the mortality and/or moribund status of the animals. According to (OECD annex 2d), a single dosage of 2000 mg/kg body weight (BW) of the PHF extract was given to the female rats. The LC_50_ or mean lethal dose value will be determined on the basis of the principle that at least two doses result in mortality rates higher than 0% and lower than 100%. [23,24].

### Sub-chronic oral toxicity

The sub-chronic toxicity research adhered to OECD guideline 408. Rats aged 8–9 weeks, 35 male and 35 female, were randomly divided into one control group and four treatment groups. The treatment groups were as follows: low-dose (250 mg/kg BW), middle-dose A (500 mg/kg BW), middle-dose B (1000 mg/kg BW), and high-dose (2000 mg/kg BW) group. Animals were weighed individually, and doses were calculated. The liver function test (LFT) was carried out at different doses of PHF extracts to analyze aspartate transaminase (AST), alanine aminotransferase (ALT), alkaline phosphatase (ALP), gamma-glutamyl transferase (GGT), cholesterol, total bilirubin, total protein, albumin, globulin and kidney function test (KFT), for creatinine, blood urea nitrogen (BUN) was performed during initiation of the study, 45th day, 91st day and 120th day. In a manner similar to that described above, laboratory tests for biochemical and hematological parameters were conducted. For 90 days, the PHF was administered once daily by oral gavage between 9 and 10 AM. All rats were monitored for symptoms of toxicity, morbidity and death throughout the trial. Weekly measurements of both food intake and body weight were taken. Before and after the investigation, ophthalmological evaluations were conducted. Weekly neurobehavioral observations were conducted. At the conclusion of the treatment, all rodents underwent clinical pathology evaluations. After 91 days of the investigation, macroscopic and histopathological examinations of the vital organs were conducted, and absolute organ weight was also calculated [25]. The data were presented as the mean ± standard deviation. Using two-way ANOVA and Tukey's multiple comparison test, the mean difference between all groups was analysed. The confidence interval was established at 95% and the p-value was modified to 0.05. For both genders, the control group and other dosing groups were compared with determine significance. The baseline hematology and clinical chemistry values for Swiss Albino Wistar rodents served as a normal value reference for the hematology and biochemistry examination results [26].

#### Clinical pathology

Blood samples were taken from the rodent before the study began, on the 45th day of the study, and at the conclusion of the treatment. The blood samples were collected from rodents under mild isoflurane anaesthesia by orbital plexus puncture using a fine heparinized capillary tube. Prior to blood collection, rats were famished overnight (*ad libitum* imbibing water supply). Blood samples were drawn for hematology (vials containing 4% EDTA anticoagulant for whole blood), coagulation parameters (vials containing 3.2% sodium citrate anticoagulant for plasma separation), and biochemical analysis (vials containing no anticoagulant for serum separation).

#### Pathological intervention

Under the supervision of a veterinary pathologist, all rodents were asphyxiated with carbon dioxide and then subjected to a complete comprehensive necropsy. All rodents were thoroughly inspected for external defects. The cranial, thoracic and abdominal cavities were cut and opened, and the organs were thoroughly examined for abnormalities. All gross abnormalities noted during the necropsy were documented. Organs and tissues were collected, weighed and preserved from both sexes. Trimming was used to remove adhering adipose tissue from the organs, and the organs' moist weights were recorded. All organs were preserved in a 10% formalin solution that was neutrally buffered. The paired organs were weighed collectively, and the resulting weights was adduced. After fixation, the parathyroid and pituitary weights of the thyroid gland were determined. As a percentage of total body mass, the organ weight ratio was defined. Organs and tissues of rodents from the control group and the high-dose group were preserved and examined histopathologically. The samples of vital organs and tissue were processed, embedded, sectioned to a thickness of 3–5 m, and stained with hematoxylin and eosin.

The acute and subchronic toxicity studies were conducted in accordance with the ‘Guidelines for Laboratory Animals Facility’ issued by the Committee for the Purpose of Control and Supervision of Animal Experiments in India (CPCSEA). Observance of these guidelines ensured that the animals were treated humanely throughout the experiment. In addition, it improved the welfare of animals, thereby enhancing the quality of the results of the experiments designed to advance human and animal-relevant biological knowledge. The initiative proposals were authorized by the ‘Institutional Animal Ethics Committee (IAEC)’. SRM/IAEC/2021/471 was the proposal number for both the acute and subchronic investigations.

### Anti-inflammatory study using RAW 264.7 cell line

#### Maintenance of cell lines

Raw 264.7 (mouse monocyte/macrophage cell line) is obtained from the NCCS in Pune, India. The RAW 264.7 cells were maintained in DMEM high glucose medium supplemented with 10% FBS, 1% antibiotic–antimycotic solution and 1% L-Glutamine (200 mM) in a 5% CO_2_, 18–20% O_2_ atmosphere at a temperature of 370 °C and sub-cultured every 2 days in a CO_2_ incubator. The 28th verse was used for this analysis [27].

### Tablet treatment to LPS-induced Raw 264.7 cells

Briefly, 0.5 × 106 cells/ml of Raw 264.7 were cultured in a six-well plate and incubated for 24 h to promote cell attachment and achieve the desired cell density. Induce inflammation in Raw 264.7 cells with 1 µg/ml for 2 h, then expose the cells to various concentrations of diclofenac or tablet and incubate for 24 h. Cells treated with LPS alone served as the positive or disease control, while untreated cells served as the control. Medium by itself is used as a neutral control in all ELISA investigations. All treatment groups were extracted into 15 ml centrifuge tubes, and cell lysates were frozen at -20 °C for later use.

#### Lipoxygenase (Lox) inhibition assay

A Lox inhibition assay was performed as per the method developed by Axelrod *et al* [28] with minor modifications. The 2 ml of Reaction mixture contained 50 μl of cell lysate, 200 μl of Sodium linoleate, and the remainder was made up with Tris-HCl buffer (pH-7.4). The mixture was then incubated for 30 min at 37 °C. After 30 min have passed since the formation of 5-hydroxyeicosatetraenoic acid, transfer 200 l of each reaction mixture to a 96-well plate and measure the absorbance at 234 nm using a microplate reader (ELX-800, BioTek).

% Lox inhibition was calculated using below formula:

$$\frac{\text{Absorbance of disease control}-\text{Absorbance of Test}}{\text{Absorbance of disease control}}\times 100$$

### Statistical analysis

The data were presented as the mean and standard deviation of the cohort. Student's *t*-test or analysis of variance (ANOVA) with Tukey's multiple comparisons were used to determine the significance of body weight, food consumption, absolute organ weight, relative organ weight, hematology and clinical chemistry. Statistical significance was set at p < 0.05.

## Results

### Acute oral toxicity assessment

**Figure 1.**
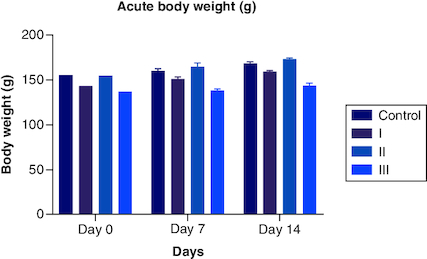
Acute body weight of female rats monitored for up to 14 days per OECD guidelines 423, showing no significant difference from start to end of the study.

During the assessment for the acute toxicity study, all the animals were carefully monitored for any signs and symptoms of the development of toxicity at the first 30 min, 1 h and 24 h. They were continued every day for 14 days. The animal body weights were monitored daily, and the mean body weight was calculated for each week. In a preliminary study with a dose of 2000 mg/kg, there were no indications of toxicity. The body weight of each rat before receiving polyherbal formulation, after 7 days, and after 14 days was compared (Table 1 & Figure 1), and their graphs showed no significant difference (p < 0.05) in body weight. The weight gain for the week 1 and 2 during the 14-day observations was also analyzed and showed no prominent changes. Table 1 shows the behavioral parameters of the female Wistar rat during the observational period, where the animals showed normal signs after the dose. However, the water consumption was less in three animals when compared with the control group.

**Table 1.** Mean body weight variation for acute study (weight in grams (g)).

Animal	Day 0	Day 7	Day 14
Control	155.5 ± 0	160.42 ± 2.32	168.7 ± 1.66
1	143.3 ± 0	151 ± 2.39	159.2 ± 1.55
2	155 ± 0	164.7 ± 4.29	173.3 ± 1.38
3	137 ± 0	138.38 ± 1.9	143.78 ± 2.95

The macroscopic examination of the heart, liver, lung, stomach, pancreas, kidney, brain and spleen revealed that there were no abnormalities noted. The absolute organ weight showed that the polyherbal formulation didn't show any significant effect on their organ weight. The histopathological examination of the organs mentioned above showed no significant changes or abnormalities detected in any of the organs. Delayed death was also not noticed during the 14 days of observation, and the animals remained active until the end of the study. As there was no mortality involved, LC_50_ was not calculated. The globally harmonized system (GHS) classification criteria for acute toxicity suggested that this polyherbal formulation was classified in category 5 or unclassified, i.e. (>2000).

### Sub-chronic toxicity assessment

The animals were carefully monitored during the sub-chronic study for any signs of abnormal behavior, changes in their eating pattern, body weight variations and primarily any signs of toxicity. The animals, both male and female, did not reveal any abnormal effects and no death in the animals during the 90-day study period.

Hematologic examinations for the hemoglobin level, total red blood cell (RBC), hematocrit, mean corpuscular volume (MCV), mean corpuscular hemoglobin (MCH), mean corpuscular hemoglobin concentration (MCHC), platelet, mean platelet volume (MPV), red cell width (RDW), total white blood cell (WBC), eosinophils, basophils, neutrophils, lymphocytes, and monocytes in WBC differential are presented at Table 4. The hematological parameter in the male and female rat groups, which received the polyherbal extracts before and after the 45th day, showed no significant changes when compared with the control animal, except there was a slight reduction in RBC level at a dose of 250 mg/kg from the original range in both the genders (7.17 ± 0.67) (7.07 ± 0.43) (p < 0.05) when compared with the control (7.58 ± 0.54). The reduction in RBC level could not be critically analyzed in that particular group of animals that received 250 mg/kg. At first, both the male and female showed a reduction in packed cell volume (PCV) in the group receiving 250 mg/kg (43.12 ± 1.27) (43.34 ± 0.97) (p < 0.05) of PHF extract and mean corpuscular volume (MCV) in the group receiving 250 mg/kg (54.8 ± 2.14) (54.58 ± 0.64) (p < 0.05) and 2000 mg/kg (54.18 ± 1.64) (53.98 ± 0.49) (p < 0.05) at the 45th day of the test when compared with that of control (44.2 ± 1.72) continued in Table 3. Similarly, the hematological data on the 91st day and 120th day showed no significant changes in the genders when compared with the controls, except reduction in MCV in only male groups receiving 2000 mg/kg (54.8 ± 0.42) (p < 0.05) dose at 91st day of the test to that of control (58.27 ± 2.17). All hematological parameters showed average values.

**Table 2.** Mean body weight variation for sub-chronic study.

Dose mg/kg	Week 0		Week 1		Week 2		Week 3		Week 4^§^		Week 5^§^		Week 6^§^	
	M^§^	F	M^§^	F	M^§^	F	M	F	M	F	M	F	M	F
Control	143 ± 11.2	117.2 ± 12.1	151 ± 8.73	121.4 ± 7.58	173 ± 11.3	125.6 ± 14.7	214 ± 11.67	131.3 ± 8.61	244 ± 9.87	133.6 ± 12.4	255 ± 10.3	141 ± 13.2	262 ± 9.82	143 ± 8.78
250	191.4 ± 7.34	137.4 ± 6.32	208 ± 7.59	144.4 ± 8.34	213.6 ± 8.9	145.8 ± 8.3	224.4 ± 9.81^ns^	141.2 ± 9.43	223 ± 7.39	152.6 ± 12.1	227.2 ± 10.4	156.8 ± 8.44	215.2 ± 9.43	148.6 ± 7.43
500	152.4 ± 8.46^ns^	112.8 ± 7.87	152.8 ± 6.9^ns^	109.8 ± 6.83	172.8 ± 11.3^ns^	126.6 ± 11.4	182.2 ± 10.6^§^	145.6 ± 7.65	204.6 ± 8.32	137.8 ± 11.3	226.6 ± 8.46	136.6 ± 8.38	231 ± 12.12	133.4 ± 6.84
1000	190 ± 8.54	147.8 ± 11.73	197.4 ± 13.08	158.2 ± 12.75	202.4 ± 13.4	160 ± 13.6	208.2 ± 13.46^ns^	155 ± 6.37	217.6 ± 6.68	159 ± 11.45	233.5 ± 7.84	169.4 ± 12.4	237.2 ± 13.6	176.8 ± 8.67
2000	170.6 ± 9.7	144.6 ± 8.06	187.2 ± 8.7	164.6 ± 11.4	182 ± 8.5^ns^	163.2 ± 6.4	196 ± 9.81^‡^	161 ± 9.43	212.1 ± 11.9	178.4 ± 9.37	221.1 ± 7.64	187.6 ± 8.74	233.4 ± 11.4	184 ± 14.7

Dose mg/kg	Week 7		Week 8^§^		Week 9		Week 10^§^		Week 11^§^		Week 12^§^		Week 13^§^	
	M^§^	F^§^	M	F	M^§^	F^§^	M	F	M	F	M	F	M	F
Control	267 ± 8.67	145 ± 9.87	275 ± 9.34	147 ± 12.3	272 ± 11.2	155 ± 7.34	287 ± 5.6	159 ± 9.4	292 ± 7.7	164.4 ± 9.8	304 ± 12.6	174.5 ± 11.4	298.6 ± 6.7	173.6 ± 9.4
250	235.8 ± 13.4	156.2 ± 6.74	238.8 ± 11.3	159.4 ± 11.71	253.5 ± 8.6	150 ± 6.5	255.4 ± 8.5	153.2 ± 9.6	257.3 ± 8.6	156.4 ± 11.5	261.3 ± 8.37	159.3 ± 12.7	264.2 ± 12.1	161.5 ± 11.2
500	248.8 ± 8^‡^	140.6 ± 9.13	237.4 ± 12.5	152.2 ± 10.2	243.4 ± 9.3	150.7 ± 7.3	245.7 ± 9.8	152.2 ± 10.3	245.3 ± 6.4	154.5 ± 8.4	254 ± 7.48	156.3 ± 8.67	255.3 ± 11.1	156.2 ± 6.8
1000	244.2 ± 11.3	177.2 ± 7.48	254.6 ± 9.68	183.2 ± 10.4	256 ± 11^†^	171.2 ± 10.6	256.3 ± 7.4	172.1 ± 8.7	258.3 ± 7.3	174.3 ± 7.67	261.2 ± 12.1	176.1 ± 11.4	262.3 ± 9.3	176.4 ± 6.7
2000	243 ± 14.3	186.2 ± 9.32	247.6 ± 9.37	185.6 ± 9.88	244.2 ± 11.3	182.3 ± 9.4	247.3 ± 6.8	182.5 ± 8.4	251.2 ± 11.2	186 ± 6.78	254.3 ± 14.6	184.6 ± 12.3	257.3 ± 8.7	187.6 ± 10.3

Data shown as mean± SD (n = 20). Statistical significance shown from control to other groups (weeks) for both the male and female denoted by.

† (<0.01).

‡ (<0.001).

§ (<0.0001).

The p-value 0.05 was significant from week 4 to week 13 when compared with the control group in both the gender and the adjusted p-value was <0.0001 with 95%^§^ CI.

**Table 3.** Significant changes of hematological parameters of wistar rats orally administered with PHF for 90-days and a 30-day post treatment recovery studies.

Days	Dose (mg/kg)	Total WBC 10^3^ Cu.mm		Ne%		Ly%		Mo%		Eo%		Ba%		RBC Million/Cu.mm		Hb g/dl	
		M	F	M	F	M	F	M	F	M	F	M	F	M	F	M	F
Day 0	Common	9.07 ± 0.99	8.57 ± 0.29	19.1 ± 0.94	19.05 ± 0.85	75.36 ± 0.91	74.05 ± 3.95	4 ± 0.08	3.9 ± 0.12	2 ± 0.01	1 ± 0.2	1 ± 0.02	0	7.58 ± 0.54	7.33 ± 0.04	14.4 ± .57	13.7 ± .52
Day 45	Control	9.65 ± 0.84	9.78 ± 0.48	21.1 ± 0.34	18.75 ± 0.58	81.47 ± 1.09	76.32 ± 1.84	4 ± 0.03	2.8 ± 0.02	2 ± 0.06	0	0	0	8.28 ± 0.14	7.43 ± .05	14.8 ± .62	14.2 ± .62
	250	9.9 ± 0.18	9.54 ± 0.63	18 ± 1.2	19.12 ± 0.67	75.51 ± 2.51	75.51 ± 1.46	4 ± 0.04	3 ± 0.04	2 ± 0.01	0	0	0	**7.17 ± 0.67** ^§^	**7.07 ± 0.4** ^§^	14.1 ± 0.49	14.1 ± 0.32
	500	10.45 ± 0.74	9.78 ± 0.89	15 ± 0.98	19.01 ± 1.21	76 ± 1.21	75.45 ± 0.91	3 ± 0.03	3 ± 0.06	1 ± 0.04	0	0	0	7.92 ± 0.6	7 ± 0.65	14.31 ± 0.96	13.41 ± 0.74
	1000	10.7 ± 0.54	10.74 ± 0.6	17 ± 1.86	19.45 ± 1.24	80.51 ± 3.1	78.51 ± 1.1	3 ± 0.01	4 ± 0.02	1 ± 0.02	0	0	0	8.01 ± 1.07	7.31 ± 0.61	14.06 ± 0.87	13.86 ± 0.42
	2000	11.7 ± 0.9	10.67 ± 0.3	20 ± 1.56	19.08 ± 0.87	86.5 ± 4.31	80.5 ± 2.31	3 ± 0.04	4 ± 0.01	1 ± 0.01	1 ± 0.01	0.8 ± 0.01	0	8.25 ± 0.56	7.45 ± 0.59	14.6 ± 0.54	14.16 ± 0.71
Day 91	Control	10.7 ± 1.46	9.37 ± 0.09	19.26 ± 1.24	18.64 ± 0.66	82.36 ± 0.9	75.24 ± 1.2	3 ± 0.02	2.1 ± 0.2	1 ± 0.02	1 ± 0.03	0.7 ± 0.04	0	8.32 ± 0.67	7.67 ± 1.04	15.2 ± .32	14.7 ± 0.41
	250	10.9 ± 0.64	9.34 ± 0.41	18.32 ± 0.21	19.02 ± 0.47	75.42 ± 1.01	75.5 ± 0.32	4 ± 0.04	4 ± 0.04	2 ± 0.03	0	0	0	**7.16 ± 0.67** ^§^	7.87 ± 0.43	14.87 ± 0.31	14.12 ± 0.21
	500	10.51 ± 0.04	9.31 ± 1.34	16.04 ± 0.46	19.71 ± 0.31	75.87 ± 0.46	75.14 ± 0.42	3 ± 0.03	2 ± 0.03	1 ± 0.02	0	0	0	8.07 ± 0.83	7.96 ± 0.42	14.36 ± 0.54	13.67 ± 0.46
	1000	10.4 ± 0.34	10.02 ± 0.2	17.03 ± 1.86	19.16 ± 0.37	77.47 ± 2.01	77.92 ± 0.61	3 ± 0.01	3 ± 0.02	1 ± 0.03	0	0	0	8.16 ± 0.62	8.01 ± 0.41	14.16 ± 0.27	13.92 ± 0.25
	2000	11.3 ± 0.06	10.97 ± 0.3	19.01 ± 0.49	18.06 ± 0.47	82.15 ± 1.01	81.35 ± 0.46	3 ± 0.04	4 ± 0.01	1 ± 0.01	1 ± 0.01	0.8 ± 0.01	0.4	8.25 ± 1.23	7.84 ± 0.07	14.91 ± 0.32	14.34 ± 0.82
Day 120#	Control	10.4 ± 0.72	8.76 ± 0.76	20.07 ± 0.68	18.73 ± 0.42	79.76 ± 0.87	74.74 ± 0.84	3 ± 0.02	2.3 ± 0.62	2 ± 0.01	1 ± 0.01	0	0	8.18 ± 0.54	7.33 ± .04	14.98 ± 0.36	14.2 ± 0.33
	250	9.9 ± 0.18	9.54 ± 0.24	18.01 ± 0.02	18.62 ± 0.44	75.47 ± 1.04	74.89 ± 0.63	4 ± 0.04	3 ± 0.04	2 ± 0.01	0	0	0	7.47 ± 1.33	7.42 ± 0.32	14.54 ± 0.34	13.98 ± 0.46
	500	9.32 ± 0.74	9.36 ± 0.39	15.47 ± 0.43	19.31 ± 1.21	75.67 ± 0.31	75.36 ± 0.47	3 ± 0.03	2 ± 0.01	1 ± 0.02	0	0	0	7.97 ± 0.41	7.96 ± 0.65	14.24 ± 0.62	13.41 ± 0.54
	1000	9.67 ± 0.54	10.14 ± 0.4	17.32 ± 0.46	19.22 ± 0.34	77.31 ± 1.41	77.97 ± 1.07	3 ± 0.01	3 ± 0.02	1 ± 0.02	0	0	0	8.06 ± 0.47	7.88 ± 0.47	14.56 ± 1.67	13.82 ± 0.28
	2000	10.87 ± 0.9	10.27 ± 0.1	20.09 ± 0.3	19.36 ± 0.46	81.75 ± 2.53	79.15 ± 0.46	3 ± 0.04	4 ± 0.01	1 ± 0.01	1 ± 0.01	0.8 ± 0.01	0	8.12 ± 0.64	7.47 ± 0.03	14.62 ± 0.36	14.23 ± 0.82
**Ref. Range**		**7.2–12.6 10^3^ Cu.mm**		**6–27%**		**66–91%**		**1–4%**		**<4%**		**<1%**		**7.21–8.45 million/Cu.mm**		**13.2–16.4 g/dL**	

Days	Dose (mg/kg)	PCV%		MCV fl		MCH pg/ml		MCHC g/dL		RDW%		MPV fl		PLT 10^3^ Cu.mm			
		M	F	M	F	M	F	M	F	M	F	M	F	M	F		
Day 0	Common	44.2 ± 1.72	43.65 ± 0.91	58.27 ± 2.17	57.34 ± 1.07	18.3 ± 0.85	17.9 ± 0.73	33.3 ± 0.84	31.6 ± 0.76	14.42 ± 1.62	13.21 ± 0.72	7.17 ± 0.75	6.4 ± 0.37	933.2 ± 95.41	850 ± 52.3		
Day 45	Control	44.2 ± 0.79	43.75 ± 0.04	59.34 ± 1.06	58.62 ± 0.09	19.72 ± 1.5	18.3 ± 0.67	32.8 ± 0.63	32.4 ± 0.44	13.42 ± 0.73	14.2 ± 0.64	8.17 ± 0.32	7.4 ± 0.46	1033.2 ± 65.3	950 ± 32.3		
	250	**43.12 ± 1.27** ^¶^	**43.34 ± 0.97** ^§^	**54.8 ± 2.14** ^‡^	**54.58 ± 0.64** ^‡^	19.1 ± 0.58	18.81 ± 1.4	32.46 ± 0.87	32.32 ± 0.46	11.3 ± 0.64	12.13 ± 0.4	7.4 ± 0.03	6.9 ± 0.14	1069 ± 21.54	989 ± 18.64		
	500	43.71 ± 0.65	43.65 ± 0.32	55.8 ± 1.85	55.98 ± 0.65	18.1 ± 1.23	18.3 ± 1.64	32.63 ± 1.43	32.45 ± 0.39	13.3 ± 0.59	12.8 ± 0.62	7.2 ± 0.07	6.8 ± 0.17	1048 ± 33.65	1048 ± 13.45		
	1000	44.23 ± 0.74	43.72 ± 0.69	55.8 ± 0.87	55.81 ± 0.34	18.8 ± 1.07	18.7 ± 1.04	33.43 ± 1.54	33.49 ± 0.74	14.6 ± 0.83	13.26 ± 0.3	7.3 ± 0.23	6.7 ± 0.63	1198 ± 25.31	1098 ± 28.41		
	2000	44.17 ± 0.53	44.26 ± 0.03	**54.18 ± 1.64** ^‡^	**53.98 ± 0.49** ^‡^	18.8 ± 1.26	18.7 ± 0.86	32.63 ± 1.42	32.23 ± 0.31	14.7 ± 0.56	13.77 ± 0.42	7.4 ± 0.34	6.7 ± 0.46	1194 ± 28.63	1164 ± 21.33		
Day 91	Control	44.2 ± 0.93	44.45 ± 0.72	59.64 ± 1.4	57.34 ± 1.07	18.3 ± 0.85	17.9 ± 1.73	33.3 ± 0.84	31.6 ± 0.76	13.42 ± 0.73	14.2 ± 0.64	8.73 ± 1.47	7.34 ± 0.6	1001.6 ± 35.3	951 ± 28.7		
	250	43.62 ± 0.62	43.64 ± 1.27	58.18 ± 1.07	56.67 ± 0.37	18.31 ± 0.64	18.62 ± 0.3	32.22 ± 1.46	32.31 ± 1.21	11.3 ± 0.98	12.43 ± 1.14	7.46 ± 1.83	6.91 ± 1.37	1069 ± 22.62	989 ± 18.64		
	500	43.71 ± 0.41	43.75 ± 0.82	55.91 ± 0.55	56.08 ± 0.05	18.38 ± 0.52	18.2 ± 0.64	32.51 ± 1.74	32.43 ± 1.47	13.3 ± 1.59	12.18 ± 1.41	7.26 ± 1.37	6.8 ± 0.46	1042 ± 23.41	1037 ± 13.45		
	1000	44.36 ± 0.52	43.87 ± 0.87	55.68 ± 0.47	55.84 ± 0.67	18.62 ± 0.47	18.7 ± 0.36	33.41 ± 1.62	33.46 ± 1.21	14.7 ± 1.23	13.36 ± 1.13	7.31 ± 0.68	6.7 ± 1.34	1187 ± 19.24	1100 ± 26.37		
	2000	44.26 ± 0.87	44.34 ± 0.63	**54.8 ± 0.42** ^§^	55.81 ± 0.64	18.73 ± 0.38	18.7 ± 0.54	32.42 ± 1.49	32.63 ± 0.91	14.7 ± 1.16	13.47 ± 0.61	7.14 ± 0.86	6.7 ± 0.38	1162 ± 38.41	1161 ± 20.68		
Day 120	Control	45.2 ± 1.72	43.67 ± 0.56	59.47 ± 0.47	57.34 ± 1.07	18.62 ± 0.43	18.1 ± 0.41	33.3 ± 0.84	31.6 ± 0.76	13.02 ± 0.95	13.89 ± 0.71	8.49 ± 0.67	7.9 ± 0.37	1061.2 ± 65.3	968 ± 36.21		
	250	43.61 ± 1.12	43.57 ± 1.38	58.06 ± 0.81	56.67 ± 0.37	18.31 ± 0.64	18.62 ± 0.3	32.22 ± 1.46	32.31 ± 1.21	11.3 ± 0.98	12.43 ± 1.14	7.46 ± 1.83	6.91 ± 1.37	1069 ± 22.62	989 ± 18.64		
	500	43.66 ± 0.98	43.77 ± 0.13	55.78 ± 0.37	56.08 ± 0.05	18.38 ± 0.52	18.2 ± 0.64	32.51 ± 1.74	32.43 ± 1.47	13.3 ± 1.59	12.18 ± 1.41	7.26 ± 1.37	6.8 ± 0.46	1042 ± 23.41	1037 ± 13.45		
	1000	44.47 ± 1.37	43.41 ± 1.21	55.68 ± 0.47	55.84 ± 0.67	18.62 ± 0.47	18.7 ± 0.36	33.41 ± 1.62	33.46 ± 1.21	14.7 ± 1.23	13.36 ± 1.13	7.31 ± 0.68	6.7 ± 1.34	1187 ± 19.24	1100 ± 26.37		
	2000	44.14 ± 0.92	44.65 ± 1.14	55.8 ± 0.61	55.81 ± 0.64	18.73 ± 0.38	18.7 ± 0.54	32.42 ± 1.49	32.63 ± 0.91	14.7 ± 1.16	13.47 ± 0.61	7.14 ± 0.86	6.7 ± 0.38	1162 ± 38.41	1161 ± 20.68		
**Ref. Range**		**43.6–48.6%**		**55.8–62.2 fl**		**17.7–20.1 pg/ml**		**31.4–33.6 g/dL**		**10.5–14.9%**		**6.2–9.8 fl**		**250–1200 10^3^ Cu.mm**			

Data shown as mean± SD (n = 20). Statistical significance shown from control to other biochemical parameters at different doses for both the male and female denoted by:

† Values are indicated in mean ±SD^#^.

‡ <0.01.

§ <0.001.

¶ <0.0001.

# The p-value 0.05 was significant from week 4 to week 13 when compared with the control group in both the gender and the adjusted p-value was <0.0001 with 95% ^¶^CI.

WBC-White Blood Cells, Ne-Neutrophils, Ly-Lymphocytes, Mo-Monocytes, Eo-Eosinophils, Ba-Basophils, RBC-Red Blood Corpuscles, Hb-hemoglobin.

PCV: Packed Cell Volume; MCV: Mean Corpuscular Volume; MCH: Mean Corpuscular hemoglobin; MCHC: Mean Corpuscular hemoglobin Concentration; RDW: Red Cell Distribution Width; MPV: Mean Platelet Volume; PLT: Platelet count.

**Table 4.** Significant changes of Liver Function Test of wistar rats orally administered with PHF for 90-days and a 30-day post treatment recovery studies.

Day	Dose mg/kg	SGOT/(AST) U/L		SGPT/(ALT) U/L		ALP U/L		GGT U/L		Cholesterol		Total Bilirubin mg/dL		Total Protein g/dL		Albumin g/dL		Globulin g/dL	
		M	F	M	F	M	F	M	F	M	F	M	F	M	F	M	F	M	F
Day 0	Common	64.5 ± 0.67	62.4 ± 0.32	23.2 ± 0.12	24.1 ± 0.54	97.5 ± 2.1	97.2 ± 1.3	7.2 ± 0.13	6.8 ± 0.35	62 ± 1.32	62 ± 0.39	0.3 ± 0.1	0.3 ± 0.1	6.5 ± 0.3	6.3 ± 0.4	4.1 ± 0.1	4.1 ± 0.1	4.1 ± 0.1	4.1 ± 0.1
Day 45	Control	65.3 ± 0.67	65.3 ± 0.67	22.1 ± 0.32	22.1 ± 0.47	98 ± 1.2	98 ± 1.3	8.2 ± 0.11	8.2 ± 0.37	67 ± 1.45	67 ± 1.47	0.3 ± 0.1	0.3 ± 0.1	6.7 ± 0.4	6.6 ± 0.3	4 ± 0.2	4 ± 0.2	4 ± 0.1	4.3 ± 0.2
	250	69.1 ± 0.63	67 ± 0.47	27.6 ± 0.17	27.6 ± 0.38	89 ± 2.3	89 ± 1.6	7.6 ± 0.23	7.6 ± 0.29	68 ± 1.32	68 ± 1.64	0.3 ± 0.21	0.3 ± 0.21	6.9 ± 0.1	6.9 ± 0.6	4.2 ± 0.1	4.2 ± 0.1	4.3 ± 0.3	4.2 ± 0.1
	500	76.4 ± 0.94	74.4 ± 0.94	27.4 ± 0.14	27.3 ± 0.36	93 ± 2.2	93 ± 2.3	6.4 ± 0.64	6.3 ± 0.48	66 ± 1.45	66 ± 1.32	0.3 ± 0.13	0.3 ± 0.13	7.2 ± 0.2	7.1 ± 0.3	4.2 ± 0.1	4.2 ± 0.2	5.2 ± 0.3	4.2 ± 0.1
	1000	74.2 ± 0.47	72.2 ± 0.47	26.7 ± 0.21	26.6 ± 0.84	99 ± 1.8	99 ± 2.6	5.3 ± 0.76	5.3 ± 0.43	61 ± 2.32	61 ± 2.12	0.4 ± 0.21	0.4 ± 0.21	7 ± 0.3	6.9 ± 0.3	4.1 ± 0.2	4.1 ± 0.1	3.8 ± 0.6	4.1 ± 0.2
	2000	68.1 ± 0.82	69.1 ± 0.82	24.4 ± 0.24	24.2 ± 0.47	110 ± 6.3	110 ± 5.1	4.7 ± 0.62	4.7 ± 0.39	68 ± 1.74	68 ± 1.56	0.4 ± 0.13	0.4 ± 0.13	6.4 ± 0.2	6.2 ± 0.4	4 ± 0.2	4.3 ± 0.2	4.1 ± 0.4	4.3 ± 0.2
Day 91	Control	68.1 ± 0.64	67.1 ± 0.64	21.3 ± 0.14	21.2 ± 0.36	124 ± 4.8	118 ± 3.5	8.1 ± 0.16	8 ± 0.24	64 ± 1.28	64 ± 1.07	0.39 ± 0.1	0.39 ± 0.1	6.5 ± 0.1	6.5 ± 0.2	4 ± 0.3	4.1 ± 0.3	3.7 ± 0.3	4.1 ± 0.3
	250	78.6 ± 0.73	77.6 ± 0.73	23.2 ± 0.18	23.3 ± 0.36	**157 ± 3.7** ^†^	**151 ± 3.2^†^**	9.3 ± 0.14	9 ± 0.31	65 ± 1.39	65 ± 1.24	**0.58 ± 0.1** ^‡^	**0.57 ± 0.1** ^‡^	6.9 ± 0.2	6.8 ± 0.3	4 ± 0.2	4.4 ± 0.1	4.3 ± 0.6	3.8 ± 0.2
	500	72.6 ± 0.38	70.6 ± 0.38	24.1 ± 0.21	24.2 ± 0.46	123 ± 4.3	127 ± 3.4	**3.4 ± 0.21** ^‡^	**3.4 ± 0.37** ^‡^	54 ± 1.27	53 ± 1.31	0.54 ± 0.1	0.55 ± 0.1	6.8 ± 0.2	6.8 ± 0.4	4 ± 0.1	4.5 ± 0.2	5.4 ± 0.1	4 ± 0.1
	1000	67.5 ± 0.46	64.5 ± 0.46	27.6 ± 0.12	27.5 ± 0.53	126 ± 1.7	121 ± 2.6	4.6 ± 0.12	4.6 ± 0.46	51 ± 1.56	51 ± 1.43	0.34 ± 0.1	0.34 ± 0.1	6.9 ± 0.3	6.7 ± 0.3	3.8 ± 0.1	4.3 ± 0.2	4.7 ± 0.1	4 ± 0.2
	2000	61.4 ± 0.28	64.4 ± 0.28	21.7 ± 0.27	21.6 ± 0.62	**129 ± 2.3** ^†^	121 ± 1.4	5.2 ± 0.17	5.2 ± 0.32	58 ± 2.12	57 ± 2.38	0.54 ± 0.1	0.55 ± 0.2	6.6 ± 0.1	6.4 ± 0.3	4 ± 0.2	4.1 ± 0.3	4.6 ± 0.2	4 ± 0.2
Day 120	Control	67.7 ± 0.67	66.7 ± 0.67	23.4 ± 0.64	23.1 ± 0.19	122 ± 1.7	122 ± 1.7	7.5 ± 0.43	7.5 ± 0.37	69 ± 2.37	68 ± 1.04	0.41 ± 0.2	0.41 ± 0.3	6.1 ± 0.1	6 ± 0.25	4 ± 0.1	4 ± 0.2	4.2 ± 0.2	4.2 ± 0.1
	250	74.2 ± 0.66	71.2 ± 0.66	18.7 ± 0.17	18.4 ± 0.24	110 ± 2.1	109 ± 1.3	6.8 ± 0.32	6.9 ± 0.63	68 ± 1.43	66 ± 1.51	0.46 ± 0.1	0.4 ± 0.12	6.3 ± 0.1	6.3 ± 0.2	4 ± 0.3	4.3 ± 0.2	4.1 ± 0.3	4 ± 0.1
	500	66.2 ± 0.52	68.2 ± 0.52	18.9 ± 0.16	18.2 ± 0.72	102 ± 1.2	100 ± 2.4	6.6 ± 0.12	6.6 ± 0.57	57 ± 1.67	55 ± 1.32	0.42 ± 0.2	0.41 ± 0.2	6.1 ± 0.2	6.1 ± 0.3	4 ± 0.2	4.4 ± 0.2	3.9 ± 0.1	4 ± 0.1
	1000	61.5 ± 1.32	63.5 ± 1.32	19.7 ± 0.24	19.5 ± 0.42	100 ± 2.3	101 ± 1.7	5.5 ± 0.18	5.4 ± 0.72	48 ± 1.46	47 ± 1.24	0.31 ± 0.1	0.31 ± 0.1	6.1 ± 0.1	6.1 ± 0.1	3.9 ± 0.2	4.2 ± 0.1	4.1 ± 0.1	4 ± 0.2
	2000	58.7 ± 0.74	57.7 ± 0.74	19.2 ± 0.38	18.8 ± 0.84	98 ± 1.1	96 ± 1.4	4.9 ± 0.16	4.8 ± 0.23	62 ± 1.67	59 ± 1.31	0.4 ± 0.14	0.43 ± 0.1	5.9 ± 0.1	5.9 ± 0.1	4 ± 0.1	4.1 ± 0.2	4.3 ± 0.2	4.1 ± 0.2
**Reference**		**45.7–80.8 U/L**		**17.5–30.2 U/L**		**56.8–128.0 U/L**				**50–100 mg/dL**		**0.2 - 0.55 mg/dl**		**5.6–7.6 g/dl**		**3.8–4.8 g/dL**		**1.5–5.5 g/dL**	

All the values are indicated in mean ±SD. Data shown as mean± SD (n = 20). Statistical significance shown from control to liver profile at different doses for both the male and female denoted by:

† <0.01.

‡ <0.001.

ALP: Alkaline Phosphatase; ALT: Alanine Transaminase; AST: Aspartate Aminotransferase.

In the liver function test results, there was no statistically significant difference between the groups and the control group from day 1 to day 45 and from day 120 during recovery. There was a modest rise in ALP (157 ± 3.7) (151 ± 3.2) (p < 0.05) and total bilirubin (0.58 ± 0.1) (0.57 ± 0.1) (p < 0.05) in both sexes after 91 days of therapy, as shown by LFT data. There were also modest variations in ALP between the control group (124 ± 4.8) and the male group receiving 2000 mg/kg (129 ± 2.3). The data in Table 4 displays normal ranges for all parameters. This result showed that liver function in rats was unaffected by doses of up to 2000 mg/kg given over 91 days.

The examination of KFT during the sub-chronic toxicity study was conducted by examining the BUN and creatinine levels of plasma from day 0 to post-recovery. It showed that no significant differences were noted in all groups from the day of initiation until the day of the recovery period compared with that of the control group. However, there was a slight reduction in BUN (p < 0.05) in a male group receiving a 2000 mg/kg dose. All the parameters showed normal values, and data is tabulated in Table. These findings revealed that rats on doses up to 2000 mg/kg for 91 days didn't affect rat renal functions – data in Table 5.

**Table 5.** Renal/kidney function test of wistar rats orally administered with PHF for 90-days and a 30-day post treatment recovery studies.

Days	Dose	BUN^ns^		Creatinine^ns^	
		M	F	M	F
Day 0	Common	18.32 ± 2.34	18.64 ± 2.07	0.7 ± 0.03	0.6 ± 0.03
Day 45	Control	11.57 ± 1.44	11.37 ± 2.34	0.61 ± 0.02	0.63 ± 0.01
	250	14.61 ± 3.12	14.61 ± 3.12	0.79 ± 0.04	0.74 ± 0.04
	500	14.49 ± 2.87	14.49 ± 2.87	0.75 ± 0.05	0.72 ± 0.05
	1000	13.41 ± 1.62	13.41 ± 1.62	0.83 ± 0.07	0.73 ± 0.07
	2000	10.74 ± 1.19	**9.54 ± 1.09** ^†^	0.87 ± 0.07	0.78 ± 0.07
Day 91	Control	17.21 ± 1.67	17.21 ± 1.67	0.65 ± 0.07	0.61 ± 0.07
	250	14.48 ± 3.28	14.48 ± 3.28	0.79 ± 0.06	0.79 ± 0.06
	500	12.61 ± 2.46	12.61 ± 2.46	0.75 ± 0.03	0.68 ± 0.07
	1000	10.61 ± 4.18	10.61 ± 4.18	0.83 ± 0.02	0.8 ± 0.06
	2000	10.74 ± 3.32	10.74 ± 3.32	0.87 ± 0.03	0.84 ± 0.04
Day 120	Control	18.46 ± 2.48	18.46 ± 2.26	0.62 ± 0.06	0.6 ± 0.06
	250	12.48 ± 2.34	12.48 ± 3.14	0.79 ± 0.02	0.79 ± 0.02
	500	11.61 ± 3.4	11.4 ± 2.44	0.75 ± 0.01	0.72 ± 0.01
	1000	10.61 ± 4.1	10.1 ± 2.67	0.83 ± 0.06	0.81 ± 0.06
	2000	11.28 ± 1.4	11.28 ± 1.61	0.87 ± 0.05	0.82 ± 0.05
**Ref.**		**10.00–33.00 mg/dl**		**0.5–2.2 mg/dl**	

All the values are indicated in mean ±SD (n = 20). The p-value 0.05 was non-significant when compared with the control group in both BUN and creatinine from day 0 to day 120.

† The levels of blood urea nitrogen were dropped to low in female taking 2000 mg/kg of PHF tablet.

**Figure 2.**
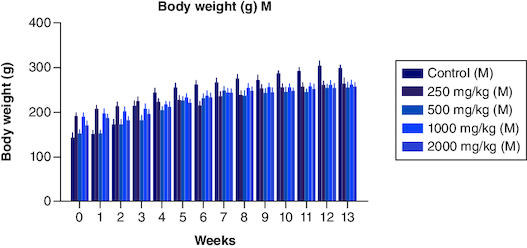
Body weight variation in males from a period of 0th week to 13th weeks after the dosage of PHF tablet. Body weight variation in females from period of 0th week to 13th weeks after the dosage of PHF tablet.

**Figure 3.**
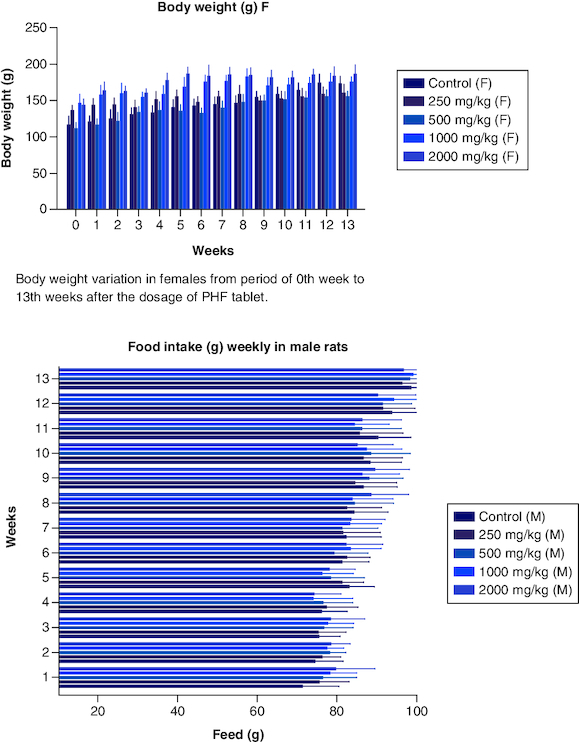
Food intake variation in males from period of 0th week to 13th weeks after the dosage of PHF tablet. Food intake variation in females from period of 0th week to 13th weeks after the dosage of PHF tablet. Mean absolute organ weight in males after dosage of PHF tablet. Mean absolute organ weight variation in females after dosage of PHF tablet.

The body weight of the male groups from 250 mg, 500 mg, 1000 mg, and 2000 mg showed less significant differences when compared with that of the control male group (p < 0.05) from the 0th to the 3rd week. Not much significant difference was noted from weeks 4th, 5th, 6th,7th,9th,10th, 12th and 13th weeks. However, body weight decreased progressively from week 5 to week 13, although not substantially, compared with weight gain in the male control group. Overall, the treated male groups showed less increase in their body weight when compared with the control male group (Figure 2). In the female group, the body weight showed non-significant differences from 0 weeks to the 3rd and 6th week at 250 mg/kg dose when compared with that of the control group in females. However, much significant difference was noted from the 4th week to the 13th week compared with that of the control group in females (Figure 3). The body weight was higher in the treated group (p < 0.05) when compared with that of the control group in females. The CI was 95%, and the adjusted p-value for significant was <0.0001. Data is presented in Table 2.

From the very beginning of the trial, investigators kept a close eye on the average amount of food both male and female rats consumed. Male participants in the control group ate more than their counterparts in the other groups did. The mean food intake of male rats in all groups which received polyherbal formulation at a dose of 250, 500, 1000 and 2000 mg/kg between the first and fourth weeks did not show any significant difference (p < 0.05) when compared with that in the control group. However, from week 5, there was less consumption of food in a dose-dependent manner when compared with that of the control group. Similarly,the food intake in female groups differs from that of the male group. In weeks 1 to 3 researchers observed little significant difference (p < 0.05) when compared with that of the control group. The consumption of food was invariable with the dose-dependent group and that of the control. The food intake was high in the 12th and 13th week in both the male and females. However, Tukey's multiple comparison tests revealed (p < 0.05) that none of the groups, male and female, differed significantly from the control group. There was no consistent pattern of decreased or increased food consumption after administration of the polyherbal formulation, as seen by the data, which revealed that food intake was either greater or lower than in the control group. However, there was high consumption of water in all the groups were observed. Throughout the studies, we found no signs of diarrhoea or constipation in the feces of either the treatment or control groups. Data is presented in Table 6.

**Table 6.** Food intake of Wistar rats post dosage for over a period of 90 days (week 0 to week 13).

Dose (mg/kg)	Week 1^ns^		Week 2^ns^		Week 3^ns^		Week 4^ns^		Week 5^ns^		Week 6^ns^		Week 7^ns^	
	M	F	M	F	M	F	M	F	M	F	M	F	M	F
Control	71.4 ± 9.1	73.4 ± 8.7	74.6 ± 6.99	73.8 ± 7.58	75.6 ± 5.2	74.3 ± 4.7	76.2 ± 6.4	73.4 ± 7.2	83.2 ± 6.2	82 ± 9.1	81.4 ± 6.7	81.6 ± 8.4	82.4 ± 8.7	82.4 ± 8.9
250	75.7 ± 7.34	74.8 ± 6.32	76.4 ± 4.59	74.6 ± 8.34	75.4 ± 6.9	75.5 ± 8.3	77.5 ± 7.8	75.6 ± 6.3	81.4 ± 5.3	84.4 ± 9.3	82.6 ± 5.8	78.4 ± 7.6	81.6 ± 9.3	79.3 ± 9.4
500	76.5 ± 8.46	75.6 ± 4.87	78.3 ± 3.94	76.3 ± 6.93	76.8 ± 7.3	77.3 ± 9.8	75.4 ± 8.6	75.5 ± 8.6	78.5 ± 8.4	83.4 ± 9.4	79.4 ± 8.4	76.2 ± 10.3	81.4 ± 8.9	76.8 ± 8.7
1000	78.4 ± 6.54	76.1 ± 9.73	77.6 ± 4.08	77.8 ± 8.75	77.8 ± 6.4	78.1 ± 3.6	74.2 ± 9.8	78.3 ± 8.3	76.4 ± 7.7	75.4 ± 8.5	83.5 ± 7.6	78.5 ± 8.1	83.4 ± 7.8	77.4 ± 10.8
2000	79.8 ± 9.73	76.3 ± 8.06	78.6 ± 4.74	76.9 ± 11.4	78.5 ± 8.5	77.6 ± 5.4	74.4 ± 6.6	81.4 ± 9.4	78.2 ± 6.4	82.3 ± 10.3	82.4 ± 9.13	78.3 ± 7.82	83.7 ± 8.4	80.4 ± 8.6

**Dose mg/kg**	**Week 8 ^ns^**		**Week 9 ^ns^**		**Week 10 ^ns^**		**Week 11 ^ns^**		**Week 12 ^ns^**		**Week 13 ^ns^**			
	**M**	**F**	**M**	**F**	**M**	**F**	**M**	**F**	**M**	**F**	**M**	**F**		
Control	84.4 ± 8.4	83.4 ± 9.4	86.7 ± 8.46	84.6 ± 10.3	88.4 ± 7.8	85.6 ± 8.8	90.4 ± 8.12	86.3 ± 8.4	92.4 ± 9.2	91.7 ± 10.3	98.7 ± 10.6	92.1 ± 8.4		
250	82.6 ± 8.6	83.6 ± 8.9	84.6 ± 10.4	85.8 ± 9.87	86.7 ± 9.7	86.2 ± 7.9	85.8 ± 10.8	85.2 ± 8.6	89.2 ± 10.4	89.4 ± 9.1	96.4 ± 8.37	93.4 ± 9.7		
500	84.5 ± 9.7	84.4 ± 9.7	88.2 ± 8.4	83.5 ± 9.32	88.6 ± 9.8	84.7 ± 10.5	86.4 ± 9.74	86.4 ± 8.7	89 ± 9.34	89.4 ± 10.4	98.4 ± 7.48	96.7 ± 8.67		
1000	83.7 ± 10.4	87.3 ± 8.3	86.4 ± 9.3	86.8 ± 8.87	87.5 ± 8.8	86.5 ± 9.6	84.5 ± 8.76	87.7 ± 8.6	93.4 ± 10.9	92.2 ± 9.78	99.2 ± 9.1	98.7 ± 10.4		
2000	88.7 ± 9.3	84.7 ± 9.4	89.6 ± 8.6	85.2 ± 8.91	90.3 ± 10.1	86.7 ± 9.7	86.4 ± 9.81	86.4 ± 8.8	90.3 ± 9.5	88.4 ± 10.6	96.8 ± 10.6	99.3 ± 9.3		

Data presented in grams (g).

† The p-value 0.05 was non-significant when compared with the control group in both the genders. All the values are indicated in mean ± SD.

After 91 days of therapy, there were no gross abnormalities in any organs checked in either the treatment or control groups of both genders. There were no statistically significant changes (p < 0.05) between the treatment groups and the control group when comparing the absolute organ weight of the lungs, stomach, intestines, kidneys, brain and spleen in either the male or female groups (Table 7). Examination of internal organs following treatment of polyherbal formulation tablets at dosages up to 2000 mg/kg once a day for 91 days showed no alterations after successful consumption of PHF tablets for 90 days. The relative organ weight was not calculated as it can affect the variability of the data (Stevens 1976). Each organ was prepped for histological testing so that microscopic inspection could be performed. From research on the sub-chronic oral toxicity of a polyherbal formulation and a control group of rats (Table 7), we see light microphotographs of the transverse sections of the important organs. The standard structure, absence of cell structure modification and absence of any unfavorable effect in organs were all revealed by histopathological analysis of the treatment and control groups of rats under a light microscope with varied magnification powers. Based on these results, there appears to be no toxicity to the organs tested when tablets containing a polyherbal mixture are taken daily for 91 days.

**Table 7.** Absolute Organ weight wistar rat post sub-chronic toxicity study.

Dose (mg/kg)	Heart (g) ^ns^		Liver (g) ^ns^		Lung (g) ^ns^		Stomach (g) ^ns^		Pancreas (g) ^ns^		Kidney (g) ^ns^		Brain (g) ^ns^		Spleen (g) ^ns^	
	M	F	M	F	M	F	M	F	M	F	M	F	M	F	M	F
Control	0.52 ± 0.03	0.4 ± 0.02	2.54 ± 0.5	2.47 ± 0.4	0.86 ± 0.04	0.83 ± 0.03	0.92 ± 0.4	0.9± 0.18	0.58 ± 0.3	0.43 ± 0.04	0.44± 0.06	0.42 ± 0.1	1.29± 0.4	1.25 ± 0.6	0.25 ± 0.05	0.23 ± 0.1
250	0.54 ± 0.06	0.42 ± 0.02	2.48 ± 0.6	2.45 ± 0.8	0.84 ± 0.05	0.86 ± 0.07	0.8± 0.6	0.9 ± 0.6	0.65 ± 0.04	0.42 ± 0.06	0.53 ± 0.05	0.58 ± 0.03	1.53± 0.8	1.75 ± 0.4	0.27 ± 0.2	0.23 ± 0.06
500	0.53 ± 0.08	0.45 ± 0.01	2.53 ± 1.2	2.48 ± 0.9	0.86 ± 0.03	0.82 ± 0.04	1.1± 0.4	0.89 ±0.4	0.56 ± 0.5	0.45 ± 0.5	0.45 ± 0.05	0.47± 0.05	1.35± 0.6	1.25 ± 0.7	0.22 ± 0.06	0.20 ± 0.3
1000	0.54 ± 0.01	0.41 ± 0.03	2.46 ± 0.8	2.49 ± 0.7	0.91 ± 0.06	0.84 ± 0.04	0.9± 0.4	0.9 ± 0.4	0.61± 0.08	0.43 ± 0.4	0.51 ± 0.04	0.45 ± 0.06	1.28± 0.1	1.34± 0.4	0.19 ± 0.01	0.22 ± 0.05
2000	0.52 ± 0.02	0.44 ± 0.04	2.52 ± 1.1	2.47 ± 0.8	0.87 ± 0.07	0.88 ± 0.07	0.8± 0.4	0.9 ± 0.2	0.46 ± 0.06	0.46 ± 0.07	0.46 ± 0.08	0.43 ± 0.04	1.31± 0.6	1.41± 0.5	0.17 ± 0.1	0.19 ± 0.06

† The p-value 0.05 was non-significant when compared with the control group in both the genders^†^. All the values are indicated in mean ± SD.

### LOX Inhibition assay

The results of cellular Lox enzyme inhibition study suggested that tablet effectively inhibited the Lox enzyme expression on dose dependent manner similar to the standard anti-inflammatory drug, Diclofenac with the IC_50_ values of 14 μg/ml and 39 μg/ml, respectively (Figures 2, 9 & 10) (Table 8).

**Table 8.** Overlaid tabular column represented the Lox enzyme inhibitory effect of Tablet in comparison to the Diclofenac in LPS induced Murine macrophage (Raw 264.7) cells.

Lox inhibition assay-Summary		
Culture condition	Diclofenac	Tablet
Untreated	0	0.00
LPS-1 μg	0	0.00
LPS + Test-6.25 μg	32.27	1.65
LPS + Test-12.5 μg	43.47	15.59
LPS + Test-25 μg	64.32	36.44
LPS + Test-50 μg	82.77	55.98
LPS + Test-100 μg	92.97	74.86
Cox IC_50_ conc (μg/ml)	14	39

## Discussion

NAFLD rates have been rising, mimicking those of diabetes and metabolic syndrome. NAFL is one end point on the continuum of liver disorders that includes NASH and can progress to cirrhosis and liver cancer [29]. It is not only a result of insulin resistance, but also a primary cause of insulin resistance and major noncommunicable diseases (NCDs). The close association between NAFLD and visceral obesity obscures the role of visceral adiposity-related fatty liver as the primary patho mechanism of insulin resistance and NCDs. Similarly, to the concept of adipokines, this limitation must be surmounted. Although the epidemiology, etiology and natural history of NAFLD have been well-studied, no authorized pharmaceutical therapy has been developed for it; as a result, only broad approaches to management have been put up. All modifiable risk factors leading to advanced NAFLD development and progression must be addressed, as must the prevention of hepatic and extra-hepatic complications. These measures include (a) adopting a healthier lifestyle to encourage weight loss through dietary and physical activity changes; (b) managing the most crucial cardiometabolic risk factors; and (d) preventing hepatic and extra-hepatic complications. Several prospective medications for the therapy of NAFLD and its advanced variants have been extensively researched in the previous decade, providing some light but also creating some shadows [30].

*In silico* techniques have proven to be the first line of defense for assessing scientific evidence without the use of actual resources. Andrographolide derived from the plant A. paniculata lacks AMES toxicity, has a human maximal tolerated dose of about 0.12 log mg/kg/day, and does not inhibit hERG-I and hERG-II. The acute oral toxicity to rats (LD_50_) was determined to be 2.16 mol/kg, and the chronic oral toxicity to rats (LOAEL) was determined to be 1 log mg/kg_bw per day. It does not cause hepatotoxicity or skin irritation. *T. pyriformis* is lethal at 0.491 log g/l, while Minnow is lethal at 1.37 log mM [31].

The polyherbal formulation comprises five plants specifically selected for their association with NAFLD, T2DM, hyperlipidemia and other obesity-related disorders. The effectiveness of this unique composition is now patented and further study on its efficacy is under process. The active ingredient in each herbal plant plays a vital role in meeting its synergistic effect on liver-related disorders, but the exact mechanism could not be postulated [32]. There are reports advocating the hepatoprotective effects of *A. paniculata* in the PHF tablet, with proposed mechanisms including protection against carbon tetrachloride (CCL4) and tert-butyl hydroperoxide (t-BHP), induction of cytochrome P450, modulation of glutathione (GSH), anti-inflammatory activity, role in the apoptosis pathway, etc., [33]. *P. niruri* and *T. arjuna* in the PHF composition were selected based on their anti-oxidant properties and anti-inflammatory properties. Although both plants have been studied for their anti-diabetic properties, insulin resistance plays a role in the formation of fatty liver. *G. glabra* has been studied on humans with a daily dose of 3.5 g and can potentially reduce body fat by inhibiting 11 11-β-hydroxysteroid dehydrogenase type-1, an NADPH-dependent enzyme in the adipose tissue [34].

Here, rats given doses of up to 2000 mg/kg of a polyherbal formulation tablet had no harm. When there is a high probability that results from such a test have direct significance for safeguarding human, animal, or environmental health, the OECD recommends using a different upper fixed-dose threshold of 5000 mg/kg. When no toxicity symptoms were observed in the preliminary trial at a dosage of 2000 mg/kg, the formulation can be considered unclassified. Because the study's maximum dose recommendation (2000 mg/kg) did not induce any toxicity signs, the LD_50_ value could not be calculated. Tablets containing a polyherbal composition were tested, and their LD_50_ value was believed to be more than 2000 mg/kg after consuming it for 90 days. From this research, we can infer that the tablets containing polyherbal formulations met the unclassified criterion.

The *P. indica* contains plumbagin, which is considered to be toxic with an oral of 65 mg/kg, this active moiety has also been reported to possess significant cytotoxic activity [35].

The compound known as glycyrrhizin, which is found in *G. glabra*, has a moderate level of toxicity. The use of these substances should be approached with care in the context of pregnancy. Glycyrrhiza glabra and its active compound glycyrrhizin have distinct cytotoxic properties against malignant cells. Hypertension and hypokalemic-induced secondary illnesses are recognised as the primary adverse effects associated with the use of licorice and glycyrrhizin. The negative effects of licorice are exacerbated by certain factors, including hypokalemia, longer gastrointestinal transit time, reduced activity of Type 2 11-beta-hydroxysteroid dehydrogenase, hypertension, anorexia nervosa, advanced age and female gender [36]. Despite the fact that the phytoconstituents present in individual plants have been studied selectively for their toxic properties, very few have been shown to produce mild to moderately toxic effects. Thus, the combination of this polyherbal tablet has been effectively investigated, revealing very minor changes in biochemical, LFT and KFT parameters, as well as no significant differences in their behavior, absolute organ weight, body weight variation, or food and water consumption.

**Figure 4.**
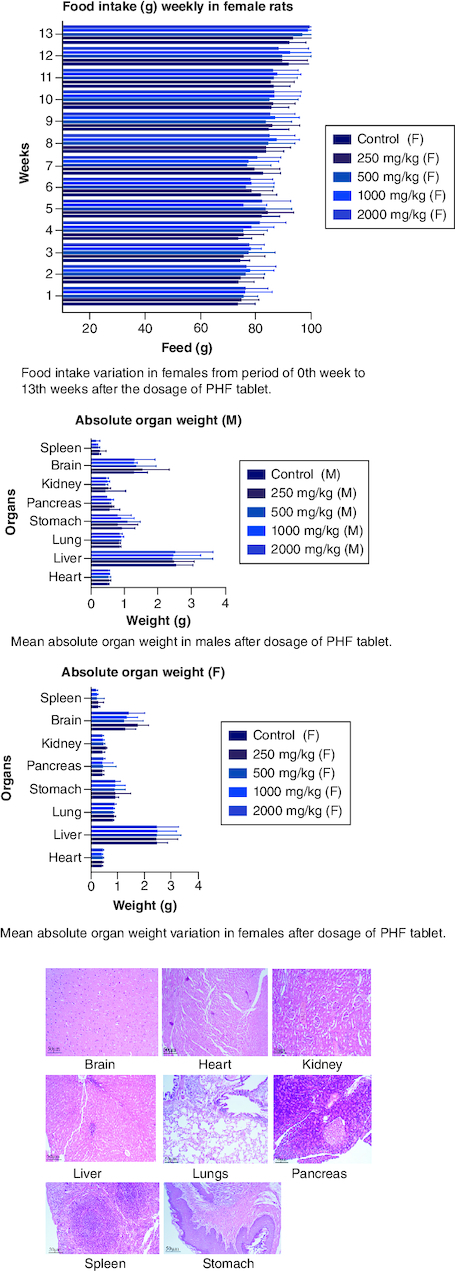
Represent the micrograph of the various vital organs from the control group.

**Figure 5.**
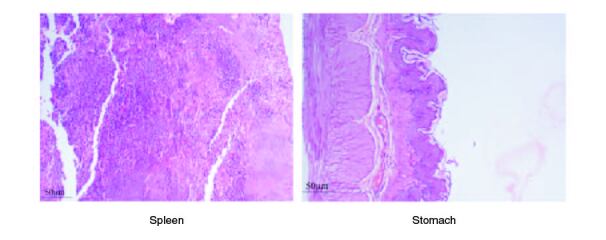
Represent the micrograph of the various vital organs from the group consuming 250 mg/kg of PHF tablet.

**Figure 6.**
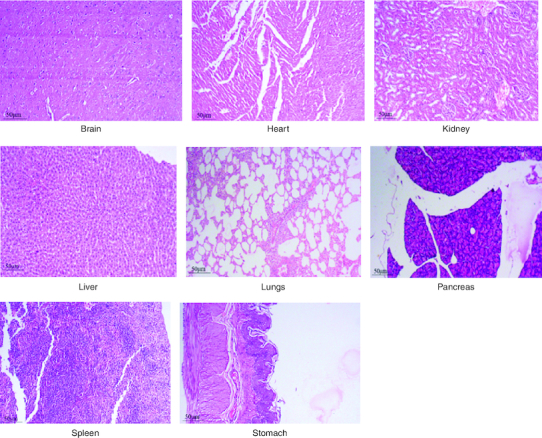
Represent the micrograph of the various vital organs from the group consuming 500 mg/kg of PHF tablet.

**Figure 7.**
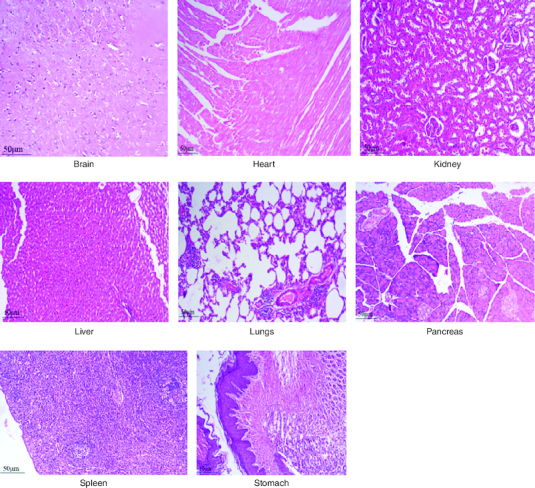
Represent the micrograph of the various vital organs from the group consuming 1000 mg/kg of PHF tablet.

**Figure 8.**
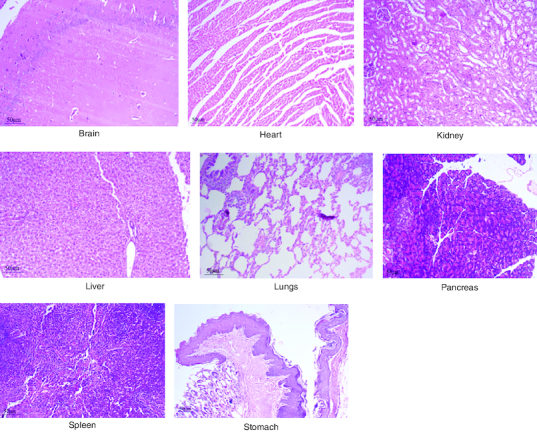
Represent the micrograph of the various vital organs from the group consuming 2000 mg/kg of PHF tablet.

Acute and chronic investigations were conducted on the freshly developed polyherbal tablet to identify the presence of any adverse component. For up to 14 days after receiving 2000 mg/kg, no major adverse effects were seen in the acute phase; thus, our PHF tablet has fallen under category 5/unclassified as per GHS and is safer up to the dose of 2000 mg/kg. The oral sub-chronic toxicity study is a test to detect toxic effects that occurred after the oral administration of testing materials in animals for a part of the animal's lifetime but not more than 10% of their lifetime. In these studies, the hematological, LFT and KFT showed mild significant changes that cannot be analyzed due to the very near effect on the parameters. The body weight and the food intake were also non-significant after the intake of PHF for over 90 days, as per OECD. The microscopic pathological examinations revealed there was one common among all the groups, including the control group, that showed there was mild multifocal hydropic degeneration of hepatocytes indicating there was an imbalance in the ions and fluid homeostasis may be due to excessive consumption of water by rats. The other histopathology in the successive groups showed no abnormalities detected in any of the other organs other than the liver (Figures 4–8). The results of this examination corroborate the obtained survival data, suggesting that polyherbal formulation tablets were safe. On clinical observation, laboratory and macroscopic examination of internal organs, and microscopically, there were no indications of toxicity in rats given polyherbal formulation tablets at doses up to 2,000 mg/kg for 91 consecutive days. Despite the presence of some limitations that need attention, the investigation failed to establish the molecular mechanism responsible for the alterations seen in hematocrit, GGT, and ALP levels. As this is a mixture of five different plants, we could not determine the exact mechanism causing the fluctuation in few parameters. Consequently, more comprehensive research is necessary to elucidate this mechanism and facilitate future discoveries.

**Figure 9.**
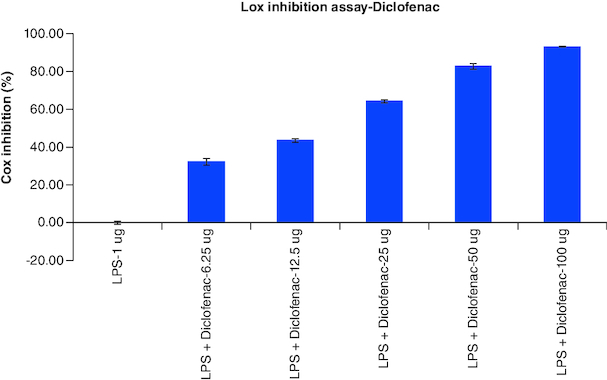
Lox enzyme levels observed in LPS alone, Diclofenac with different concentrations treatment on LPS-induced Raw 264.7 cells after the incubation period of 24 h. Each assay was performed in duplicate and the experiments were repeated once.

**Figure 10.**
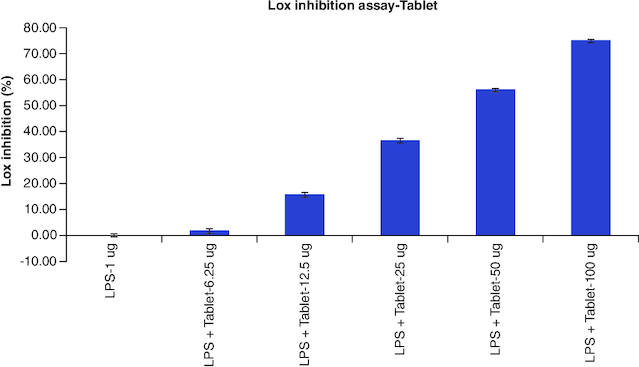
Lox enzyme levels observed in LPS alone, tablet with different concentrations treatment on LPS-induced Raw 264.7 cells after the incubation period of 24 h. Each assay was performed in duplicate and the experiments were repeated once.

## Conclusion

This study demonstrated that oral administration of an PHF tablet did not cause any fatalities, behavioral abnormalities, or significant alterations in certain biochemical and hematological markers in the rats. Alanine aminotransferase, total protein, granulocytes, and lymphocyte alterations as well as body weight reduction were noticed, nevertheless. It is evident from the study, that there is no toxicity in the animal model. For the safe exploration of its therapeutic effects, doses lower than 1000 mg/kg may be employed for future pre-clinical studies. Upon reviewing several sources of research, it has become apparent that the alterations in biochemical parameters could not be definitively attributed to a specific cause. In addition, it is necessary to use sophisticated techniques such as Flow Cytometry or Gene expression investigations by RT-qPCR in order to validate the molecular mechanism of action and ascertain alterations in biochemical markers. Consequently, more comprehensive research is necessary to elucidate this mechanism and facilitate future discoveries. Further human studies (phase I) which concentrates on the pharmacokinetics and bioavailability of the polyherbal tablet would be performed to identify the potential health implications.
